# Internetbasierte Selbsthilfe bei Einsamkeit – Eine systematische Übersicht

**DOI:** 10.1007/s00103-024-03934-1

**Published:** 2024-08-08

**Authors:** Milena Imwinkelried, Noëmi Seewer, Thomas Berger, Tobias Krieger

**Affiliations:** Institut für Psychologie, Abteilung klinische Psychologie und Psychotherapie, Universität Bern, Fabrikstrasse 8, 3012 Bern, Schweiz

**Keywords:** Subjektive soziale Isolation, Behandlungsansatz, Online, Web-basiert, Systematisches Review, Perceived social isolation, Treatment approach, Web-based, Online, Systematic review

## Abstract

**Hintergrund:**

In den letzten Jahren hat das Phänomen der Einsamkeit zunehmend Aufmerksamkeit erlangt. Einsamkeit ist weitverbreitet und kann bei längerem Anhalten negative Folgen für die psychische und physische Gesundheit haben. Internetbasierte Interventionen (IBI) zur Selbsthilfe haben sich für eine Vielzahl von psychologischen Störungen als hilfreich erwiesen. Aufgrund verschiedener spezifischer Aspekte stellen IBI auch für Einsamkeit eine vielversprechende Interventionsform dar. Ziel dieser systematischen Übersicht ist es, den aktuellen Stand der Forschung von Selbsthilfe-IBI zur Reduktion von Einsamkeit darzustellen.

**Methoden:**

Eine systematische Literaturrecherche wurde in den Datenbanken Web of Science, PubMed, Scopus, PsycInfo, MedLine, PsycIndex, Cochrane Library und PsyArXiv im Zeitraum von Dezember 2023 bis Anfang Januar 2024 durchgeführt. Eingeschlossen wurden deutsch- oder englischsprachige Originalarbeiten, die Selbsthilfe-IBI zur Reduktion von Einsamkeit untersuchten.

**Ergebnisse:**

Insgesamt konnten 8 Studien, die zwischen 2017 und 2024 publiziert wurden, in die qualitative Analyse eingeschlossen werden. Alle Studien wurden in einkommensstarken Ländern mit überwiegend gut gebildeten weiblichen Teilnehmenden durchgeführt und waren hinsichtlich ihrer internen Validität mehrheitlich zufriedenstellend.

**Diskussion:**

Die Ergebnisse der vorliegenden Übersichtsarbeit legen nahe, dass Selbsthilfe-IBI eine vielversprechende Möglichkeit zur Verringerung von Einsamkeit sein können. Die Arbeit weist jedoch auch auf weiteren Forschungsbedarf hin. Zukünftige Arbeiten sollten größere Stichproben und Menschen unterschiedlicher Altersgruppen, Geschlechter und Bildungsniveaus berücksichtigen, um die Ergebnisse der vorliegenden Übersichtsarbeit verallgemeinern zu können.

**Zusatzmaterial online:**

Zusätzliche Informationen sind in der Online-Version dieses Artikels (10.1007/s00103-024-03934-1) enthalten.

## Abstract (en)

**Background:**

Recently, the phenomenon of loneliness has received increasing attention. Loneliness is widespread and can have adverse consequences for mental and physical health if prolonged. Internet-based interventions (IBIs) for self-help have proven to be effective for a variety of psychological disorders. Due to several specific aspects, IBIs are also a relevant option for loneliness. This systematic review aims to present the current research on self-help IBIs for reducing loneliness.

**Methods:**

A systematic literature search was conducted in the databases Web of Science, PubMed, Scopus, PsycInfo, MedLine, PsycIndex, Cochrane Library and PsyArXiv between December 2023 and early January 2024. We included original German or English studies that addressed IBIs for self-help to reduce loneliness.

**Results:**

In total, eight studies published between 2017 and 2024 were included in the qualitative analysis. All studies were conducted in high-income countries, included predominantly well-educated female adults and were mostly satisfactory regarding their internal validity.

**Discussion:**

The results of this review suggest that self-help IBIs may be a promising option for alleviating loneliness. However, the work also points to the need for further research. Future studies should consider larger samples and people of different ages, genders and education levels in order to generalise the results of the present review.

## Hintergrund

Menschen fühlen sich einsam, wenn sie eine Diskrepanz zwischen den tatsächlichen und ihren gewünschten Beziehungen wahrnehmen, also wenn sie ihre aktuellen sozialen Beziehungen quantitativ und/oder qualitativ als unzureichend empfinden [1]. *Subjektive* *soziale Isolation* ist ein Synonym für Einsamkeit. Sie unterscheidet sich von *objektiver* sozialer Isolation, welche die Abwesenheit von sozialen Kontakten beschreibt [1]. So kann ein Mensch allein sein und sich nicht einsam fühlen und umgekehrt kann er von vielen Menschen umgeben sein und dennoch Einsamkeit empfinden. Eine kürzlich durchgeführte repräsentative Umfrage in der Schweiz zeigte, dass sich rund 14 % der Personen ziemlich häufig oder sehr häufig einsam fühlen [2]. Dies deckt sich mit Erhebungen aus Deutschland, in denen 10–20 % der Bevölkerung angeben, sich häufig oder ständig einsam zu fühlen [3]. Dabei ist zu beachten, dass diese Angaben möglicherweise eine Unterschätzung darstellen, da Personengruppen, die häufig von Einsamkeit betroffen sind, wie bspw. obdachlose Personen und Personen in Institutionen [4], in repräsentativen Erhebungen oft nicht erfasst werden.

Einsamkeitsgefühle können als Signal für ein unzureichend befriedigtes Bedürfnis nach sozialer Verbundenheit angesehen werden [5]. Vorübergehende Einsamkeit kann sich zwar schmerzhaft anfühlen, sie ist jedoch adaptiv, da sie zum Aufbau neuer und zur Vertiefung vorhandener sozialer Beziehungen motivieren kann [6]. Hält das Gefühl der Einsamkeit an, kann ein Teufelskreis entstehen, der die Einsamkeit über die Zeit verstärkt und aufrechterhält. Dem kognitiv-behavioralen Modell [7] zufolge können Gefühle der Einsamkeit zu einer erhöhten Sensibilität für soziale Bedrohung führen. (Chronisch) einsame Personen neigen daher eher dazu, neutrale oder ambivalente soziale Reize als bedrohlich wahrzunehmen. Dementsprechend zeigen einsame Menschen negative kognitive Verzerrungen in verschiedenen Phasen der sozialen Informationsverarbeitung, wie z. B. Aufmerksamkeits‑, Bestätigungs- und Gedächtnisverzerrungen. Diese Verzerrungen wiederum verstärken kontraproduktive soziale Verhaltensweisen, wie soziale Vermeidung, passives Verhalten oder präventive Ablehnung anderer. Dadurch erleben einsame Personen häufiger negative soziale Interaktionen, die einerseits die negativen Erwartungen bestätigen [8] und anderseits neue Einsamkeitsgefühle zur Folge haben oder diese aufrechterhalten. Infolgedessen ist es wahrscheinlicher, dass soziale Situationen auch auf biologischer Ebene mit einer Stressreaktion einhergehen, wobei sich diese u. a. negativ auf den Schlaf auswirken kann [9]. Die mit andauernder Einsamkeit einhergehende chronische Stressreaktion kann zu unterschiedlichen gesundheitlichen Problemen führen [10]. Die psychische Gesundheit kann durch (chronische) Einsamkeit beeinflusst werden, allerdings sind die Zusammenhänge komplex und nicht linear. Menschen, die unter Einsamkeit leiden, stellen eine heterogene Gruppe dar [11]. Die negativen Auswirkungen von Einsamkeit auf die Gesundheit beeinträchtigen nicht nur das individuelle Wohlbefinden, sondern sind auch mit erheblichen wirtschaftlichen Kosten für die Gesellschaft verbunden [12].

### Interventionen zur Reduktion von Einsamkeit: aktueller Stand der Forschung

(Chronische) Einsamkeit ist keine diagnostizierbare Störung. Es gibt keinen anerkannten Schwellenwert für die Ausprägung, ab welcher Einsamkeit behandlungsbedürftig ist. Der Zusammenhang zwischen Einsamkeit und verschiedenen negativen Folgen für die psychische und physische Gesundheit sowie der Leidensdruck, der mit chronischer Einsamkeit einhergeht, legt jedoch nahe, dass es wirksamer spezifischer Interventionen zur Verringerung von Einsamkeit bedarf, um Betroffene zu unterstützen [5, 10]. In Übereinstimmung mit dem kognitiven Modell der Einsamkeit [7] deuten Metaanalysen darauf hin, dass Interventionen, die auf die Veränderung maladaptiver sozialer Kognitionen abzielen, bei der Verringerung von Einsamkeit am wirksamsten sind [13, 14]. Die Ergebnisse einer neueren Metaanalyse bestätigen diese Befunde, indem sie die Wirksamkeit psychologischer Interventionen bei der Reduktion von Einsamkeit zeigen, wobei kognitiv-verhaltenstherapeutische (KVT-)Interventionen zu den effektivsten Methoden zur Verringerung von Einsamkeit gehören [15], jedoch nicht wirksamer sind als andere psychologische Interventionen [16]. Verschiedene Übersichtsarbeiten weisen zudem auf das Potenzial technologie- oder internetbasierter Interventionen zur Verringerung von Einsamkeit hin [14–17]. Dabei ist allerdings zu beachten, dass die Entwicklung und Verbreitung von evidenzbasierten Interventionen für Einsamkeit im Vergleich zu Interventionen für diagnostizierbare psychische Erkrankungen noch am Anfang stehen [18].

Bisherige Studien zu Einsamkeitsinterventionen gehen mit vielen Einschränkungen einher, wodurch die Evidenzlage eingeschränkt ist. So fokussierte die bisherige Forschung bspw. stark auf ältere Erwachsene, obwohl das Phänomen Einsamkeit über die gesamte Altersspanne hinweg verbreitet ist [16, 19]. Zudem schließen Übersichtsarbeiten, die Interventionen zur Reduktion von Einsamkeit untersuchten, oft auch Studien zu sozialer Isolation mit ein [16], obwohl Einsamkeit und soziale Isolation nicht Hand in Hand gehen müssen. Des Weiteren werden in Übersichtsstudien oft Interventionen mitberücksichtigt, die nicht speziell auf Einsamkeit ausgerichtet sind, sondern auf andere psychische oder gesundheitsbezogene Probleme abzielen [17, 18].

### Internetbasierte Selbsthilfe

Der Begriff *internetbasierte Interventionen* (IBI) wird nicht einheitlich verwendet und umfasst eine Bandbreite verschiedener Interventionsarten, von z. B. internetbasierten Selbsthilfeinterventionen über Videotherapien bis hin zu Chat-Interventionen [20]. Der Schwerpunkt des vorliegenden Übersichtsartikels liegt explizit auf IBI zur Selbsthilfe, die im Folgenden mit der Abkürzung „IBI“ gemeint sind. Dabei werden psychologische Interventionen so adaptiert, dass sie Nutzenden mittels Webseiten oder Smartphone-Apps zur Verfügung gestellt und von diesen selbstständig genutzt werden können [21]. Die Bearbeitung der Inhalte erfolgt selbstständig (ungeleitete Selbsthilfe) oder in Kombination mit kurzen Kontakten mit geschulten Personen (angeleitete Selbsthilfe). Dabei kann die Anleitung mehr oder weniger intensiv sein und telefonisch oder schriftlich erfolgen [20]. Die Anleitung dient hierbei primär der Erhöhung der Interventionsnutzung [22]. Viele IBI haben einen störungsspezifischen Fokus [21–24]. Mittlerweile gibt es aber auch einige symptomorientierte oder transdiagnostische Interventionen. Die meisten Interventionen sind in mehrere Module gegliedert, die nacheinander bearbeitet werden können. Die Vermittlung der Inhalte erfolgt vorwiegend textbasiert, wobei oft auch Audio- und Videoelemente integriert werden. Nebst psychoedukativen Teilen sind Anleitungen für Übungen, Tagebücher und Protokolle zentrale Teile solcher Interventionen. Sie können über das Programm bearbeitet und im Alltag umgesetzt werden [21]. Insbesondere angeleitete IBI zeigen bei verschiedenen psychischen Störungen vergleichbare Effektstärken wie herkömmliche Face-to-Face-Therapien [23]. Ungeleitete IBI zeigen sich ebenfalls als wirksam, in der Tendenz jedoch mit etwas geringeren Effektstärken, was u. a. mit einer höheren Abbruchrate in Zusammenhang gebracht wird [25].

Gewichtige Vorteile internetbasierter Interventionen sind z. B. die zeit- und ortsunabhängige Nutzung, die geringeren Kosten und die Skalierbarkeit, d. h. die Möglichkeit, sie vielen Menschen in gleichbleibender Qualität zur Verfügung zu stellen. Durch die hohe Anonymität können zudem die Hemmschwelle für das Aufsuchen von Unterstützung gesenkt und dadurch mehr Betroffene erreicht werden [20]. Solch niederschwellige Interventionen stellen gerade bei Beschwerden, die mit einem Stigma einhergehen, ein vielversprechendes Angebot dar, um betroffenen Personen wirksame Interventionen zugänglich zu machen.

### Die vorliegende systematische Übersichtsarbeit

In der Vergangenheit wurden verschiedene (psychologische) Interventionen zur Reduktion von belastenden Einsamkeitsgefühlen entwickelt, wobei diese sich als unterschiedlich wirksam zeigten [13, 16]. Aufgrund der stigmatisierten Natur des Phänomens scheinen IBI ein vielversprechender Ansatz zu sein [21, 26]. Die vorliegende systematische Übersichtsarbeit soll einen Überblick über den aktuellen Forschungsstand von IBI bei Einsamkeit und deren Wirksamkeit geben.

## Methoden

Die systematische Übersichtsarbeit wurde in Anlehnung an die PRISMA-Richtlinien [27] und das Cochrane Handbook [28] durchgeführt. Das Studienprotokoll wurde nicht registriert.

### Identifikation und Selektion von Studien

Die Datenbanken Web of Science, PubMed, Scopus, PsycInfo (via Ovid), MedLine (via Ovid), PsycIndex (via Ovid), Cochrane Library und PsyArXiv (via Google Scholar) wurden zwischen Dezember 2023 und Anfang Januar 2024 durchsucht. Zusätzliche Studien wurden den Bibliografien der identifizierten Studien entnommen. Eine Übersicht der Suchstrategie ist in den Onlinematerialien (Tabelle Z1) zu finden.

Studien wurden in die Analyse eingeschlossen, wenn sie nach dem Jahr 2000 veröffentlicht wurden und die folgenden Kriterien erfüllten: (1) Einsamkeit wird quantitativ als primäre Ziel- und Veränderungsvariable erfasst, (2) bei der Studie handelt es sich um eine Interventionsstudie, (3) die Studie bezieht sich auf IBI, d. h., (a) der Inhalt wird über ein internetfähiges Gerät vermittelt, (b) die Inhalte werden selbstständig ungeleitet oder angeleitet bearbeitet. (4) Bei der Studie handelt es sich um eine Primärstudie, die in englischer oder deutscher Sprache vorliegt. Detaillierte Beschreibungen der Einschlusskriterien sind in den Onlinematerialien (Tabelle Z2 und Z3) zu finden.

Als Werkzeug für die systematische Überprüfung wurde CADIMA [29] verwendet. Duplikate wurden entfernt. Die Einschlusskriterien wurden von 2 voneinander unabhängigen Gutachterinnen (MI, NS) zuerst auf die Titel und Zusammenfassungen und anschließend auf die Volltexte angewendet. Meinungsverschiedenheiten über den Einschluss von Studien wurden diskutiert. Bei Bedarf wurde mit einem dritten Reviewer (TK) Rücksprache gehalten, bis ein Konsens erzielt wurde.

### Datenextraktion und Datenanalyse

Die relevanten Daten wurden von einer Autorin (MI) extrahiert und stichprobenweise überprüft (NS). Zu den extrahierten Daten gehörten Details über die Population, die Intervention, die Studienmethoden und die Ergebnisse.

Zur Einschätzung des Risikos systematischer Verzerrungen wurde für randomisierte Studien das *Cochrane Risk of Bias 2 Tool* [30] und für nichtrandomisierte Studien das *ROBINS‑I Tool* [31] von 2 unabhängigen Reviewerinnen (MI, NS) angewandt.

## Ergebnisse

### Suchergebnisse

**Abb. 1:**
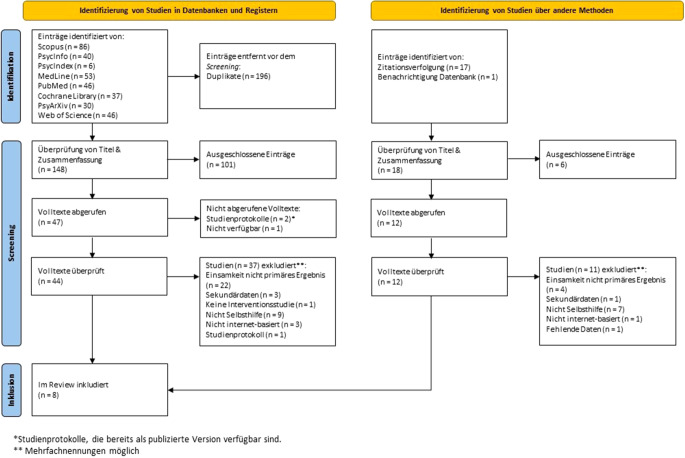
Studien-Screening-Prozess. PRISMA-Flow-Diagramm. Eigene Abbildung in Anlehnung an Page et al. [27]

Die systematische Suche in den elektronischen Datenbanken lieferte nach der Entfernung von Duplikaten 148 Treffer. Mittels der Suche von Vorwärts- und Rückwärtszitationen wurden weitere 18 Studien identifiziert. Die Prüfung aller 166 gefundenen Veröffentlichungen nach Titel und Zusammenfassung führte zu 56 Volltexten, die genauer überprüft wurden. Letztlich wurden 8 Arbeiten in die vorliegende Studie eingeschlossen [26, 32–38]. Die Einzelheiten des Studien-Screening-Prozesses können der Abb. 1 entnommen werden.

Tab. 1 fasst die Merkmale und wichtigsten Ergebnisse der einbezogenen Studien zusammen. Alle eingeschlossenen Studien wurden in Ländern mit hohem durchschnittlichen Einkommen (Schweden, *n* = 2 [26, 34]; USA, *n* = 2 [33, 37]; Schweiz, *n* = 2 [36, 38]; Australien, *n* = 1 [35]; Niederlande *n* = 1 [32]) durchgeführt und zwischen 2017 und 2024 veröffentlicht. Studienteilnehmende waren mehrheitlich Erwachsene aus der Allgemeinbevölkerung, älter als 18 Jahre (*n* = 3; [26, 34, 36]), über 50 (*n* = 1 [32]) oder über 65 Jahre alt (*n* = 2 [37, 38]). 2 Studien [33, 35] wurden mit einer Studierendenstichprobe durchgeführt. In einigen Studien [26, 34, 36, 38] wurde das Erreichen/Überschreiten eines bestimmten Schwellenwerts auf der eingesetzten Einsamkeitsskala als Einschlusskriterium für die Studie verwendet. In den meisten Studien (*n* = 7; [26, 32–34, 36–38]) waren die Teilnehmenden überwiegend weiblichen Geschlechts (44,4 % [35]–78,6 % [36]). In allen durchgeführten Studien wurde der Bildungsstand erfasst, wobei die Teilnehmenden mehrheitlich über einen Hochschulabschluss verfügten. Einsamkeit wurde in den meisten Studien (*n* = 7 [26, 33–38]) mit verschiedenen Versionen der UCLA-Einsamkeitsskala erfasst [39].

**Tab. 1:** Charakteristika der eingeschlossenen Studien

Studie (Jahr, Referenz)	Design	Stichprobenbeschreibung	Studienbedingungen	Inhalt/Beschreibung der internetbasierten Intervention	Anzahl Module/Adhärenz	Einsamkeitsmaß	Primärer Endpunkt/Follow-up	Wirksamkeit
Risk of Bias (RoB)								Effektstärke Between/Within
Bouwman et al. [32] RoB: hoch	Prä-Post	*N*: 239 Alter: M = 61,58 (SD = 7,15), Spannweite = 50–86 Weiblich: 78 % Art: Erwachsene ab 50 Jahren, die ihre Freundschaften verbessern möchten	I_1_: FEP voll-leicht^d^ (*n* = 131) I_2_: FEP leicht-voll^d^ (*n* = 108) K: –	Selbstgeleitete Online-Intervention, Adaption des „Friendship enriching program“ (FEP), 3 Bewältigungsstrategien zur Überwindung der Einsamkeit: (1) Aufbau eines Netzwerks, (2) Anpassung der persönlichen Normen, (3) Verringerung der Bedeutung der Diskrepanz zwischen tatsächlichen und gewünschten Beziehungen	5 Keine Angaben	De Jong Gierveld Loneliness Scale (6-Items)	Interventionsende, nach 11 Wochen *Follow-up*: 1 Jahr	*Between:* Keine Kontrollgruppe In beiden Gruppen signifikante Reduktion in beiden Einsamkeitsfacetten (sozial und emotional) *Within*: I_1_: *d* = 0,34 (nach 11 Wochen) I_2_: *d* = 0,33 (nach 11 Wochen)
Brühlmann-Senecal et al. [33] RoB: moderat	RCT ^a^	*N*: 221 Alter: M_I_ = 18,68 (SD_I_ = 0,35), M_K_ = 18,69 (SD_K_ = 0,39), Spannweite = 18–19 Weiblich: 59,3 % Art: Studierende im ersten Semester	I: „Nod“-Selbsthilfe-App (*n* = 100) K: Warteliste (*n* = 121)	3 Hauptkomponenten (Anwendung spezifiziert fürs Campus-Leben): (1) soziale Herausforderungen: Vorschläge für die Kontaktaufnahme mit anderen, (2) Reflexionen: kurze In-App-Übungen (soziale Erfahrungen verarbeiten und Selbstkritik abbauen), (3) schriftliche Erfahrungsberichte anderer Studierender	Keine Angaben M_I_: 36,69 (Seiten von insg. 102)	UCLA Loneliness Scale (8-Items)	Interventionsende, nach 4 Wochen *Follow-up*: 1 Monat	*Between:* Keine signifikanten Unterschiede zwischen den Gruppen *Within:* I: *d* = 0,48 K: *d* = 0,42
Dworschak et al. [38] RoB: moderat	RCT ^a^	*N*: 36 Alter: M_I_ = 70,83 (SD_I_ = 5,50), M_K_ = 72,06 (SD_K_ = 6,28), Spannweite = 65–87 Weiblich: 72,2 % Art: ältere Menschen (> 65 Jahre), die sich einsam fühlen ^c^	I: ungeleitete Selbsthilfe „NümEinsam“ (*n* = 18) K: Warteliste (*n* = 18)	Ungeleitetes Programm mit fiktionalem Coach, basierend auf KVT, mit Fokus auf kognitiver Umstrukturierung und Elementen aus der positiven Psychologie sowie der Lebensrückblick-Therapie	7 M_I_: 4,28	UCLA Loneliness Scale (9-Items)	Interventionsende, nach 7 Wochen *Follow-up*: –	*Between:* *d* = 0,29 (nicht signifikant) *Within:* I: *d* = 0,64 K: *d* = 0,82
Käll et al. [34] RoB: moderat	RCT ^a^	*N*: 73 Alter: M = 47,20 (SD = 17,63) Weiblich: 71,2 % Art: Erwachsene, die sich einsam fühlen ^c^	I: angeleitete Selbsthilfe (*n* = 36) K: Warteliste (*n* = 37)	Psychoedukation, funktionelles Verhaltensmodell, Identifizierung von Zielen, Werten, Überprüfung von dysfunktionalen Gedanken und Überzeugungen, Strategien zur Reduzierung des Grübelns, Verhaltensexperimente, Verhaltensaktivierung	8 M_I_: 4,89 Alle Module: 33,0 %	UCLA Loneliness Scale (20-Items)	Interventionsende, nach 8 Wochen *Follow-up*: 2 Jahre [42]	*Between:* *d* = 0,77 *Within*: I: *d* = 1,47 K: *d* = 0,40
Käll et al. [26] RoB: gering	RCT	*N*: 170 Alter: M = 47,50 (SD = 16,40) Weiblich: 75,9 % Art: Erwachsene, die sich einsam fühlen ^c^	I_1_: geleitete Selbsthilfe, basierend auf KVT (*n* = 68) I_2_: geleitete Selbsthilfe, basierend auf IPT (*n* = 68) K: Warteliste (*n* = 34)	KVT : Vgl. Käll et al. (2020) mit einem zusätzlichen Modul zu sozialen Fertigkeiten IPT: Assessment-Phase – Ermittlung der Einsamkeitserfahrungen, Fokus (Konflikt, Trauer, zwischenmenschliche Defizite und Rollenwechsel) auswählen. Fokus-Phase – emotionale Verarbeitung und Förderung verbundener Gefühle	9 M_I1_: 6,85 Alle Module _I1_: 55,9 % M_I2_: 6,93 Alle Module _I2_: 61,8 %	UCLA Loneliness Scale (20-Items)	Interventionsende, nach ca. 9 Wochen *Follow-up*: 4 Monate	*Between*: I_1_ vs. K: *d* = 0,71 I_1_ vs. I_2_: *d* = 0,53 I_2_ vs. K: *d* = 0,18 IPT nicht signifikant besser als K und signifikanter Unterschied zu KVT *Within*: I_1_: *d* = 1,17 I_2_: *d* = 0,63 K: *d* = 0,27
Lim et al. [35] RoB: moderat	Prä-Post ^a^	*N*: 20 Alter: M_I1_ = 21,00 (SD_I1_ = 2,41), M_I2_ = 20,36 (SD_I2_ = 2,16), Spannweite = 18–23 Weiblich: I_1_ = 44,4 %, I_2_ = 45,45 % Art: Studierende, mit/ohne diagnostizierte/r sozialer Angst	I_1_: +Connect-App, Studierende mit sozialer Angst (*n* = 9) I_2_: +Connect-App, Studierende ohne soziale Angst (*n* = 11) K: –	Gamifizierte Selbsthilfe-App, Module mit jeweils ca. 5-minütigen Aufgaben, basierend auf positiver Psychologie, wenn möglich in Videoformat (3 Arten von Videos: geteilte Erfahrung (junge Person, die auch Einsamkeitsgefühle kennt), Expert:innen (Akademiker:in), Schauspieler:innen (die Modelle erklären))	16 Alle Module _I1_: 84,7 % Alle Module _I2_: 90,3 %	UCLA Loneliness Scale (20-Items)	Interventionsende, nach 5 Wochen (wenn App 33 Tage benutzt wurde) *Follow-up*: 3 Monate	*Between*: – *Within*: Gesamt: *d* = 0,94 I_1_: *d* = 1,20 I_2_: *d* = 1,12
Seewer et al. [36] RoB: gering	RCT	*N*: 243 Alter: M = 45,77 (SD = 14,85); Spannweite = 19–80 Weiblich: 78,6 % Art: Erwachsene, die sich einsam fühlen ^c^	I_1_: angeleitete Selbsthilfe (*n* = 98) I_2_: ungeleitete Selbsthilfe (*n* = 97) K: Warteliste (*n* = 48)	Selbsthilfe-Programm basierend auf Käll et al. (2020), mit zusätzlichen Elementen zu Achtsamkeit, Akzeptanz, Selbstmitgefühl und sozialen Fertigkeiten I_1_: erhalten wöchentlich personalisierte Nachricht/Rückmeldung von Coach I_2_: erhalten automatisierte wöchentliche E‑Mail-Nachricht	9 M_I1_: 6,77 Alle Module _I1_: 42,9 % M_I2_: 5,98 Alle Module _I2_: 39,2 %	UCLA Loneliness Scale (9-Items)	Interventionsende, nach 10 Wochen *Follow-up*: –	*Between*: I_gesamt_ vs. K: *d* = 0,57 I_1_ vs. I_2_: *d* = 0,42 I_1_ vs. K: *d* = 0,80 I_2_ vs. K: *d* = 0,34 *Within*: I_1_: *d* = 1,02 I_2_: *d* = 0,73 K: *d* = 0,28
Zarling et al. [37] RoB: kritisch	Prä-Post ^a^	*N*: 11.443 Alter ^b^: M = 70,20 (SD = 4,60); Spannweite = 66–95 Weiblich: 61,6 % Art: ältere Menschen, die sich einsam fühlen ^c^	I: ungeleitete Selbsthilfe (*n* = 529) K: Warteliste (*n* = 10.914)	Selbsthilfe-Programm, mit wöchentlicher Hausaufgabe und Kontaktmöglichkeit auf Anfrage, basierend auf Akzeptanz-Commitment-Therapie	8 Alle Module: 36,0 %	UCLA Loneliness Scale (10-Items)	Interventionsende, nach Modul 8 *Follow-up*: 1 Monat	*Between:* – *Within*: I: *d* = 0,73 (Completer Sample) K: *d* = 0,49

*d* Effektgröße Cohens *d, FEP* Friendship Enriching Program, *I* Interventionsgruppe, *IPT* interpersonale Therapie, *K* Kontrollgruppe, *KVT* kognitive Verhaltenstherapie, *M* Mittelwert, *N* Stichprobengröße, *RCT* randomisiert kontrollierte Studie, *RoB* Risk of Bias, Risiko systematischer Bias-Verzerrungen

^a^ Von der Autorenschaft als Pilot-Studie bezeichnet und durchgeführt

^b^ Angaben zum Alter beziehen sich nur auf die Interventionsgruppe, da die Angaben dazu für die Gesamtstichprobe unklar waren

^c^ Diese Studie verwendete einen Schwellenwert für Einsamkeit als Einschlusskriterium für die Intervention/Studie

^d^ FEP (voll-leicht/leicht-voll) = Interventionsbedingungen, „leicht“ stimmt inhaltlich mit „voll“ überein, beinhaltet jedoch begrenztere Informationen, Anregungen zur Reflexion und keine Aufgaben. Die „voll-leicht“-Gruppe erhielt damit erst die umfassenderen Module und danach die verkürzten. Bei „leicht-voll“ gerade umgekehrt

Bei der Mehrzahl der inkludierten Studien handelt es sich um randomisiert kontrollierte Studien (RCTs; *n* = 5 [26, 33, 34, 36, 38]), davon 2 [34, 36] mit jeweils 2 Interventionsgruppen (Tab. 1 für eine detaillierte Übersicht). Bei den restlichen Studien handelt es sich jeweils um eine randomisierte Prä-Post-Studie mit 2 Interventionsgruppen, welche sich lediglich in der Reihenfolge der Module unterschieden [32], sowie 2 nicht randomisierte Beobachtungsstudien [35, 37]. In den meisten Studien wurde eine Warteliste als Kontrollgruppe eingesetzt. Dabei wurde in 5 Studien [26, 33, 34, 36, 38] der Warteliste-Kontrollgruppe nach Abschluss der Interventionsphase Zugang zur Intervention erteilt, während in einer Studie [37] der Kontrollgruppe zu keinem Zeitpunkt eine Intervention angeboten wurde. 2 weitere Studien [32, 35] hatten keine Kontrollgruppe. Die Gesamtzahl der Teilnehmenden aller Studien betrug 1531^1^. Die Stichprobengröße in den einzelnen Studien variierte von 20 [35] bis 529^2^ [37]. Von den Studienteilnehmenden, die die Basiserhebung ausgefüllt hatten, fehlten Daten zum primären Endpunkt bei insgesamt 3,6 % [33] bis 66,7 % [37].

### Form und Inhalt der internetbasierten Selbsthilfeprogramme

Die Interventionsdauer erstreckte sich von 4 [33] bis 11 [32] Wochen. Die Anzahl der Module reichte von 5 [32] bis 16 [35], wobei diese Information bei einer Studie [33] nicht vorlag. In den meisten Fällen (*n* = 5; [26, 34, 36–38]) war vorgesehen, dass die Nutzenden ein Modul pro Woche bearbeiten.

Die Interventionen basierten auf unterschiedlichen theoretischen Grundlagen: 4 nutzten vorwiegend Techniken oder Prinzipien der KVT [26, 34, 36, 38], 2 basierten auf Ansätzen der positiven Psychologie [33, 35]. Eine Intervention war achtsamkeitsbasiert [37], eine basierte auf dem interpersonellen Therapieansatz (IPT; [26]) und eine auf einem „Freundschaftsprogramm“, in dem Copingstrategien vermittelt wurden [32].

Die IBI wurden mehrheitlich in ungeleiteter Form (*n*^3^ = 7 [32, 33, 35, 36, 38]) angeboten, in 4^3^ Fällen [26, 34, 36] in angeleiteter Form (schriftlich oder telefonisch) und in einem Fall [37] erfolgte eine Kontaktaufnahme ausschließlich auf Nachfrage. Die Bearbeitung der Module wurde heterogen berichtet und die deskriptiven Angaben dazu sind der Tab. 1 zu entnehmen.

#### Wirksamkeit der Interventionen

Im Gruppenvergleich (*Between-group*) zeigte sich mehrheitlich, dass Einsamkeit in der Interventionsgruppe verglichen mit der Kontrollgruppe [26, 33, 34, 36–38] signifikant stärker reduziert werden konnte. Die Überlegenheit der Interventions- gegenüber der Kontrollgruppe konnte in 2 Studien [33, 38]^4^ sowie in der IPT-Bedingung [26] nicht nachgewiesen werden. Die Zwischengruppeneffekte lagen hierbei im kleinen bis oberen mittleren Bereich zwischen *d* = 0,34 [36] und *d* = 0,77 [34]. In einer Studie, in der ungeleitete und angeleitete Selbsthilfe verglichen wurde, erwies sich die angeleitete Selbsthilfe jener ohne menschliche Begleitung, dafür mit automatisierten Rückmeldungen, als signifikant überlegen [36]. Bezüglich Effekte innerhalb der Gruppen (*Within-group*) zeigten sich in den Interventionsgruppen Effekte von *d* = 0,33 [30] bis *d* = 1,47 [34] und entsprachen kleinen bis überwiegend großen Effekten. Mit Ausnahme einer Studie [38] waren die Effekte innerhalb der Interventionsgruppen größer als innerhalb der Kontrollbedingungen (Tab. 1).

### Risiko systematischer Verzerrungen

**Abb. 2:**
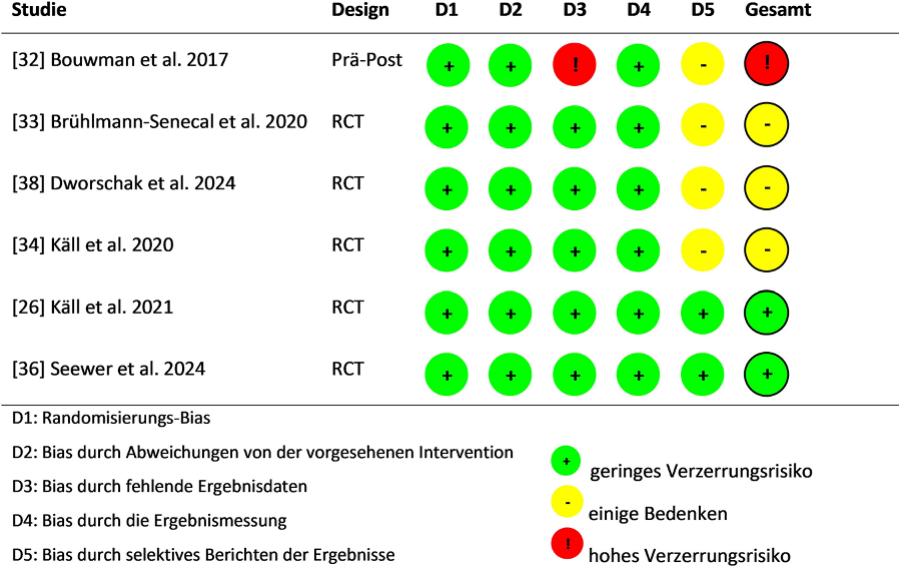
Randomisierte Studien: Bewertung des Risikos für systematische Verzerrung. Eigene Abbildung nach Higgis et al. [28]

**Abb. 3:**
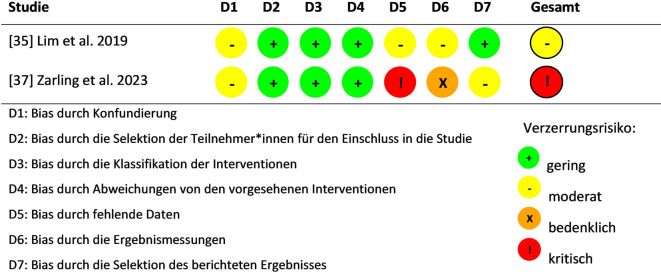
Nichtrandomisierte Studien: Bewertung des Risikos für systematische Verzerrung. Eigene Abbildung nach Higgis et al. [28]

Die methodische Qualität der eingeschlossenen Studien ist in den Abb. 2 und Abb. 3 dargestellt. Bei den randomisierten Studien [26, 32–34, 36, 38] war die häufigste Ursache für Verzerrungen die selektive Berichterstattung. Die Bewertungen für die selektive Berichterstattung über die Ergebnisse sind jedoch mit Vorsicht zu interpretieren, da für viele Studien keine Studienprotokolle verfügbar waren. Diese Studien wurden daher mit „keine Informationen“ bewertet, was zu einer negativen Beurteilung dieser Domäne führte. Die Bewertung für ein hohes Verzerrungsrisiko resultierte in der betreffenden randomisierten, nichtkontrollierten Studie [32] aufgrund der Unklarheit im Berichten und den Analysen der Ergebnisse. Die 2 nichtrandomisierten Studien [35, 37] wurden mit dem ROBINS‑I [31] anhand von 7 Domänen bewertet (Abb. 3). Die Verzerrungen kamen aufgrund von Konfundierung, fehlenden Daten, durch die Ergebnismessungen und die Selektion der berichteten Ergebnisse zustande.

## Diskussion

In dieser systematischen Übersichtsarbeit wurden internetbasierte Selbsthilfeinterventionen (IBI) zur Reduktion von Einsamkeit hinsichtlich ihres Inhalts und ihrer Wirksamkeit untersucht. Dabei interessierte v. a. der aktuelle Stand der Forschung, welche Interventionen in welchen Stichproben und mit welcher Wirksamkeit bereits untersucht wurden.

### Zusammenfassung der Ergebnisse

Die frühe Phase der Erforschung von IBI zur Reduktion von Einsamkeit spiegelt sich in den eingeschlossenen Studien wider. Alle inkludierten Studien wurden in den letzten 7 Jahren veröffentlicht. Bei den meisten Studien handelt es sich um RCTs, in denen die Interventionsgruppe mit einer Wartelisten-Kontrollgruppe verglichen wurde. Insgesamt war das Risiko einer systematischen Verzerrung relativ gering, insbesondere in den randomisierten Studien, da die meisten Studien mit einem „geringen“ oder „moderaten Risiko einer Verzerrung“ eingestuft wurden.

### Heterogenität der Interventionen

In dieser systematischen Übersichtsarbeit wird deutlich, dass in den letzten Jahren verschiedene IBI zur Reduktion von Einsamkeit entwickelt worden sind. Eine gewisse Heterogenität zeigt sich bei der theoretischen Fundierung und inhaltlichen Gestaltung der Interventionen. Beispielsweise beruhen die meisten Interventionen auf den Prinzipien der KVT, während andere IBI auf Achtsamkeit und/oder auf der positiven Psychologie basieren oder Elemente davon beinhalten. Ein Programm fokussierte auf die (Wieder‑)Gewinnung von qualitativen Freundschaften sowie (der Veränderung) der damit einhergehenden Normen und Erwartungen. Die Befunde stimmen mit der bisherigen Erkenntnis überein, dass KVT der am besten erforschte Ansatz für IBI insgesamt ist [21]. Allerdings wurden auch diese Interventionen oft mit anderen Elementen, wie Selbstmitgefühl, Achtsamkeit und Lebensrückblickstherapie ergänzt, was im Sinne eines flexiblen, modularen Ansatzes psychologischer Interventionen bei Einsamkeit sinnvoll scheint [40].

### Zielpopulation

Einsamkeit ist keine diagnostizierbare Störung. In den eingeschlossenen Studien führte dies zu unterschiedlichen Einschlusskriterien. Einige Studien schlossen nur Personen ein, die einen bestimmten Schwellenwert auf der Einsamkeitsskala erreichten, andere nicht. Die teilnehmenden Personen könnten daher einen unterschiedlichen Schweregrad/Leidensdruck der Einsamkeit zu Interventionsbeginn aufgewiesen haben. Dies könnte zu Unterschieden zwischen den Studien führen, die die Effektstärke zwischen den Studien beeinflussen könnten.

### Wirksamkeit der Interventionen

Auf deskriptiver Ebene deutet die vorliegende Übersichtsarbeit darauf hin, dass IBI mit kleinen bis mittleren Effektstärken im Vergleich zu einer Warteliste-Kontrollgruppe zum Interventionsende und mit mittleren bis großen Within-group-Effektstärken im Prä-Post-Vergleich Einsamkeit effektiv reduzieren können. Bei der Interpretation der Effektstärken ist zu berücksichtigen, dass die Teilnehmenden in den verschiedenen Studien zu Beginn der Intervention einen unterschiedlichen Leidensdruck aufwiesen (vgl. Zielpopulation), was sich auf die Wirksamkeit der Intervention auswirken kann. Die Befunde weisen jedoch generell in eine vielversprechende Richtung. Allerdings drängt sich u. a. aufgrund der Heterogenität der von Einsamkeit Betroffenen sowie der Interventionen die Frage auf, welche Interventionen für welche Betroffenen wirksam sind. Beispielsweise zeigte sich in der Studie von Seewer et al. [36], dass bei 61 % der Personen, die angeleitete Selbsthilfe erhielten, und bei 53 % der Personen mit automatisierter Rückmeldung eine reliable Veränderung erfolgte (Completer-Analyse). Dies weist darauf hin, dass nicht alle Personen gleichermaßen von einer solchen Intervention profitieren können.

Bei den Personen in den eingeschlossenen Studien handelt es sich, wie in vielen Studien zu IBI, um selbstselektierte Stichproben. Dabei weisen die eingeschlossenen Studien einen sehr hohen Frauenanteil auf. Außerdem wurden in den inkludierten Studien nur erwachsene Personen untersucht, keine schloss Jugendliche ein, obschon Studien darauf hindeuten, dass Einsamkeit bei Jugendlichen und jungen Erwachsenen ein prävalentes Phänomen darstellt [19]. Die Studien mit jüngeren Teilnehmenden konnten keine Gruppenunterschiede finden [33] oder wiesen zu kleine Stichproben auf [35]. Überdies waren in allen Studien Personen mit höherem Bildungsniveau übervertreten. Eine kürzlich erschienene Metaanalyse stellte fest, dass sich Einsamkeit bei Menschen mit höherem Bildungsniveau durch die Interventionen signifikant stärker verbessern ließ [15]. Ein weiterer Hinweis darauf, dass relevante Betroffene (Personen mit geringerer Bildung, mit Migrationshintergrund, Jugendliche etc.) in den bisherigen Studien tendenziell nicht erreicht worden sind.

Was sind Merkmale von wirksamen Interventionen? Eine kürzlich erschienene Übersichtsarbeit schlussfolgerte diesbezüglich, dass sich wirksame von nicht wirksamen Interventionen anhand des Grades der Begleitung sowie des Behandlungsansatzes unterscheiden [15]. Auch für IBI weisen die Ergebnisse einer der eingeschlossenen Studien [36] darauf hin, dass eine menschliche Begleitung einer automatisierten überlegen ist. Dies steht in Einklang mit Ergebnissen zu IBI bei depressiven Symptomen, dass ein gewisses Maß an Kontakt die Motivation zur Nutzung einer Selbsthilfe-Intervention erhöht [22] und selbst eine minimale Begleitung der Adhärenz und Wirksamkeit förderlich ist [24]. In Übereinstimmung mit diesen Ergebnissen wurde in einer der untersuchten Studien [32] festgestellt, dass das Üben, z. B. mit Aufgaben, effektiver ist als die reine Darbietung von Bewältigungsstrategien. Dies deckt sich mit den Ergebnissen aus der oben erwähnten Übersichtsarbeit, dass bei ineffektiven Interventionen kaum versucht wurde, soziale Interaktionen zwischen den Sitzungen zu fördern [15]. Der Einbezug von Interaktionen zwischen den Sitzungen könnte nicht nur während der Dauer der Intervention wichtig sein, sondern auch längerfristig, indem die Inhalte auch nach dem formalen Ende der Intervention präsenter und damit besser in den Alltag integrierbar sind.

Die Effekte der meisten KVT-basierten Interventionen sowie die Überlegenheit der KVT-IBI gegenüber einer IPT-IBI [26] weisen darauf hin, dass dieser therapeutische Ansatz zur Reduktion von Einsamkeit besonders vielversprechend sein könnte. Dies unterstreicht auch die Annahmen des kognitiven Modells der Einsamkeit [7], bei dem die Veränderung maladaptiver sozialer Kognitionen ein möglicher Ansatzpunkt zur Verringerung von Einsamkeit darstellt. Allerdings wird aus der Übersichtsarbeit ersichtlich, dass es auch andere Ansätze zur Behandlung von Einsamkeit gibt, die einen gewissen Grad an Effektivität erreichen [32, 35, 37]. Insofern bedarf es eines besseren Verständnisses davon, welche Personen unter welchen Umständen von welcher Intervention profitieren können. Es ist möglich, dass auch bei IBI zu Einsamkeit Kontextfaktoren der Intervention eine zentrale Rolle spielen, wie z. B. ein klares Ziel der Intervention, Übungen für den Transfer in den Alltag oder die Art der Begleitung während der Intervention (z. B. individualisiert vs. automatisiert). Eine kürzlich erschienene Sekundäranalyse einer der eingeschlossenen Studien weist bspw. darauf hin, dass eine Übereinstimmung der Aufgaben und Ziele der Intervention mit den Bedürfnissen der Person prädiktiv für den Interventionserfolg ist [41]. Andere Faktoren, wie z. B. die Größe des sozialen Netzwerks, hatten hingegen keinen Einfluss auf das Einsamkeitserleben am Ende der Intervention. Dies deutet wiederum darauf hin, dass Einsamkeit und soziale Isolation auf unterschiedlichen zugrunde liegenden Prozessen beruhen und Interventionen entsprechend ausgerichtet werden sollten.

Über die Nachhaltigkeit der berichteten positiven Effekte lässt sich erst wenig sagen, da nur wenige Studien einen längeren Follow-up-Zeitraum betrachteten. Die bisherigen Befunde weisen in eine aussichtsvolle Richtung, so zeigt sich z. B. in einer Follow-up-Studie zur Studie von Käll et al. [32] die Tendenz, dass eine Reduktion im Einsamkeitserleben in den Interventionsgruppen über einen Zeitraum von 2 Jahren nach Interventionsende aufrechterhalten werden konnte [42].

### Stärken, Limitationen und Ausblick

Diese systematische Übersichtsarbeit zeigt den aktuellen Stand der Forschung von IBI zur Reduktion von Einsamkeit und deren Wirksamkeit auf. Eine Stärke dieser Übersichtsarbeit ist, dass sie Interventionsstudien unabhängig von ihren Designs berücksichtigt. Zudem wurde bei jeder eingeschlossenen Studie eine Qualitätsbewertung durchgeführt. Als eine Einschränkung gilt es anzufügen, dass nur englisch- und deutschsprachige Arbeiten eingeschlossen wurden, was möglicherweise dazu geführt hat, dass zusätzliche Erkenntnisse nicht berücksichtigt werden konnten.

Weitere Forschungsarbeiten sind nötig, die den langfristigen Nutzen psychologischer IBI gegen Einsamkeit analysieren und untersuchen, ob die Veränderungen nachhaltig sind. Zudem sind weitere Studien erforderlich, um besser zu verstehen, welche Interventionen für wen und unter welchen Umständen am besten geeignet sind, um Einsamkeit effektiv zu reduzieren. Ebenso erscheint es relevant, den Zusammenhang zwischen Einsamkeit und damit verbundenen psychischen Erkrankungen genauer zu betrachten und zu untersuchen. Dabei gilt es bspw. besser zu verstehen, welche Veränderungen durch welche psychologischen Interventionen adressiert werden und ob es Effekte gibt, die indirekt mit Einsamkeit zusammenhängen. Interventionen gegen Einsamkeit könnten ggf. auch präventiv dazu beitragen, dass sich psychische Erkrankungen nicht voll entwickeln. Nicht zuletzt sollten künftige Forschungsarbeiten die Wirksamkeit psychologischer und eher „sozialer“ Interventionen (z. B. soziales Verschreiben, d. h. eine Maßnahme, um Personen mit nichtmedizinischen Unterstützungsquellen in der Gemeinschaft in Verbindung zu bringen [43]) vergleichen und auch untersuchen, ob eine Kombination aus einer psychologischen und einer gemeinschaftsbasierten Intervention wirksamer ist als eine der beiden Interventionsebenen allein [18, 44]. Da es verschiedene relevante sozioökonomische und geografische Aspekte [18] gibt, die Einsamkeit beeinflussen, sind auch komplementäre Maßnahmen auf anderen Ebenen nötig, um Einsamkeit in der Gesellschaft reduzieren zu können.

## Fazit

Die vorliegende Arbeit zu IBI für Einsamkeit bietet einen systematischen Überblick über den aktuellen Forschungsstand. Es wurden nur wenige Studien gefunden, die ausschließlich in einkommensstarken Ländern mit vorwiegend gut gebildeten weiblichen Teilnehmenden durchgeführt wurden und die hinsichtlich ihrer internen Validität ansprechend waren. Zukünftige Forschung sollte sich auf die Testung in diverseren Stichproben, den sozialen Kontext der Betroffenen und die Langfristigkeit der Effekte konzentrieren. Insgesamt weist diese Übersichtsarbeit darauf hin, dass IBI einen vielversprechenden Ansatz zur Reduktion von Einsamkeitsgefühlen darstellen könnten. Weitere Forschung ist jedoch notwendig, um die Wirksamkeit zu verbessern und eine breite Anwendbarkeit zu gewährleisten.

## Supplementary Information

Tabellen Z1, Z2 und Z3

## Footnotes

1 Angaben ohne Kontrollgruppe der Studie von Zarling et al. [37], da die Angaben zur Gesamtstichprobe unklar waren.

2 Diese Zahl bezieht sich auf die Interventionsgruppe, die Angaben zur Kontrollgruppe und damit zur Gesamtstichprobe sind unklar.

3 Die Zahlen beziehen sich auf die Interventionsgruppen, von denen es pro Studie mehrere geben kann. Kontrollgruppen sind ausgeschlossen.

4 Die statistische Power war bei einer Studie [38] möglicherweise zu klein, um signifikante Effekte zu finden.
