# Supplementary material for: Internet-based self-help for loneliness—a systematic review
Source: Bundesgesundheitsblatt Gesundheitsforschung Gesundheitsschutz. 2024 Aug 8;67(10):1161–71. [Article in German] doi: 10.1007/s00103-024-03934-1 (PMC11424658; doi:10.1007/s00103-024-03934-1)
Supplement: Supplementary file 1 — Tabellen Z1, Z2 und Z3 [file 103_2024_3934_MOESM1_ESM.pdf]

Tabelle Z1. Suchstrategie zur Identifikation von Studien zu internetbasierten Selbsthilfeinterventionen für Einsamkeit

Datenbank oder zusätzliche Ressource	Suchbegriff	Resultate	Datum der Suchabfrage	Kommentar
**Scopus**	TITLE-ABS-KEY (((lonel* OR "subjective social isolation") AND ("self help" OR "self support") AND (intervention OR treatment OR program OR counsel?ing OR e-therapy OR psychoedu* OR icbt OR approach) AND (online OR internet OR web* OR mobile* OR app* OR digital OR computer))) AND PUBYEAR > 1999 AND PUBYEAR < 2024	86	09.01.24	Einschränkung in der Suche, Publikationsjahr nach 2000
**Ovid (PsycInfo)**	((lonel* or "subjective social isolation") and ("self help" or "self support") and (intervention or treatment or program or counseling or e-therapy or psychoedu* or iCBT or approach) and (online or internet or web* or mobile* or app* or digital or computer))	40	09.01.24	2000-current
**Ovid (Medline)**	((lonel* or "subjective social isolation") and ("self help" or "self support") and (intervention or treatment or program or counseling or e-therapy or psychoedu* or iCBT or approach) and (online or internet or web* or mobile* or app* or digital or computer))	53	09.01.24	2000-current
**Ovid (Psyndex)**	((lonel* or "subjective social isolation") and ("self help" or "self support") and (intervention or treatment or program or counseling or e-therapy or psychoedu* or iCBT or approach) and (online or internet or web* or mobile* or app* or digital or computer))	6	09.01.24	2000-current
**PubMed**	Search: ((lonely or loneliness or "subjective social isolation") and ("self help" or "self support") and (intervention or treatment or program or counseling or e-therapy or psychoeducation or iCBT or approach) and (online or internet or web-based or mobile or app or digital or computer)) Filters: from 2000 - 2024	46	10.01.24	Suchstring mit MeSH-Terms (automatisch von PubMed generiert)
**Cochrane library (Trials)**	((lonely or loneliness or "subjective social isolation") and ("self help" or "self support") and (intervention or treatment or program or counseling or e-therapy or psychoeducation or iCBT or approach) and (online or internet or web-based or mobile or app or digital or computer))	37	10.01.24	

**PsyArXiv**	(loneliness OR lonely OR "subjective social isolation") AND ("self help" OR "self support") AND (intervention OR treatment OR program OR counseling OR "e therapy" OR psychoeduation OR iCBT OR approach) AND (online OR internet OR "web based" OR "mobile based" OR app OR digital) computer source:PsyArXiv	30	10.01.24	gesucht über Google Scholar (Einschränkung auf diese Datenbank, da bei PsyArXiv Searchstring nicht funktioniert), ab Publikationsjahr 2000
**Web of Science**	((lonel* or "subjective social isolation") and ("self help" or "self support") and (intervention or treatment or program or counseling or e-therapy or psychoedu* or igbt or approach) and (online or internet or web* or mobile* or app* or digital or computer))	46	10.01.24	ab Publikationsjahr 2000
**Search alert**	internetbasierte Selbsthilfe Einsamkeit	1	13.12.23	Benachrichtigung von Ovid
**Citation Chaising**	Zitationsverfolgung (Reviews, Metaanalysen)	17	28.12.23	Metaanalysen und Reviews: Baker et al. 2018, Casanova et al. 2021, Chen & Schulz 2018, Chipps et al. 2017, Dworschak et al. 2022, Ibarra et al. 2021, Khosravi et al. 2016, Morris et al. 2014, Oliveira et al 2021, Osborne et al 2021, Welch et al 2023, Shah et al 2021, Morrish et al 2023

Tabelle Z2. Einschlusskriterien Titel und Zusammenfassungen

Kriterium	Kommentar, Beschreibung
Einsamkeit – primäres Outcome	Einsamkeit wird als primäres Outcome gemessen, Einsamkeit explizit erwähnt (verwandte Konstrukte wie bspw. soziale Isolation werden nicht miteinbezogen), falls unklar, ob es primäres oder sekundäres Outcome ist - entsprechend als "unclear" kodieren
Primärstudie / Studienprotokoll [1]	die Studie ist eine Primärstudie (d.h. Reviews, Metaanalysen werden ausgeschlossen), Studienprotokolle, die eine Primärstudie bspw. ein RCT beschreiben werden eingeschlossen. Falls es sich um ein Protokoll für eine Metaanalyse oder systematisches Review handelt, wird dieses ausgeschlossen.
Intervention	Es handelt sich um eine Interventionsstudie. Idealerweise mit Einsamkeit als primäres Outcome / Veränderungsvariable.
Selbsthilfe	Inhalte/Strategien werden selbst erarbeitet (alles, was grundsätzlich alleine bearbeitet werden kann). Eine motivierende Nachricht / Kontakte zum "dranbleiben" widerspricht sich nicht, solange die Inhalte nicht gemeinsam mit anderen Personen erarbeitet werden. Beim Title & Abstract Rating wird blended Therapy auch miteinbezogen.
internetbasiert	Inhalt (der Intervention) muss online/web-basiert/digital/per App/mobil vermittelt werden, damit die Studie in den Review eingeschlossen wird

Tabelle Z3. Einschlusskriterien Volltext

Kriterium	Kommentar, Beschreibung
Einsamkeit – primäres Outcome	Einsamkeit wird als primäres Outcome gemessen. Verwandte Konstrukte bspw. soziale Isolation sind abzugrenzen.
Primärstudie / Studienprotokoll [1]	Die Autorenschaft generiert selbst (Roh-)Daten. Studien, die Sekundäranalysen machen, werden ausgeschlossen.
Intervention	Versuchspersonen werden bewusst einer Bedingung (geplant & gezielt) zugeteilt.
Selbsthilfe	Inhalte werden selbst erarbeitet. Motivierende Nachrichten (jeglicher Form) von Fachpersonen oder/und Peers sind "erlaubt", der Großteil wird jedoch selbst gemacht. Bei Blended Treatments müssen Daten von einer guided/self-guided Selbsthilfe-Gruppe klar ersichtlich getrennt in der Studie aufgeführt werden (Kombinationsbehandlung online & f2f, fällt nicht in diese Kategorie). Studiendesign: Blended (internetbasiert + f2f) vs. internetbasiert werden eingeschlossen, alles muss "grundsätzlich alleine bearbeitet" werden können
internetbasiert	die Inhalte (Texte, Übungen, Videos, etc.) werden internetbasiert/digital (mobile/per App) vermittelt. telehealth, technologiegestützte Interventionen (z.B. Robotertiere, Therapie via Videocall) werden ausgeschlossen, insofern kein (oder zu vernachlässigender) Teil der Intervention nicht internetbasiert (i.e.S.) ist.

[1] Der Ausschluss von Studienprotokollen wurde post-hoc entschieden.
